# Concentration of inverted repeats along human DNA

**DOI:** 10.1515/jib-2022-0052

**Published:** 2023-07-25

**Authors:** Carlos A. C. Bastos, Vera Afreixo, João M. O. S. Rodrigues, Armando J. Pinho

**Affiliations:** DETI – Department of Electronics, Telecommunications and Informatics, IEETA – Institute of Electronics and Informatics Engineering of Aveiro, University of Aveiro, 3810-193 Aveiro, Portugal; LASI – Intelligent Systems Associate Laboratory, Aveiro, Portugal; CIDMA – Center for Research and Development in Mathematics and Applications, DMAT – Department of Mathematics, University of Aveiro, 3810-193 Aveiro, Portugal

**Keywords:** distance distribution, human genome, inverted repeats, Markov model

## Abstract

This work aims to describe the observed enrichment of inverted repeats in the human genome; and to identify and describe, with detailed length profiles, the regions with significant and relevant enriched occurrence of inverted repeats. The enrichment is assessed and tested with a recently proposed measure (*z*-scores based measure). We simulate a genome using an order 7 Markov model trained with the data from the real genome. The simulated genome is used to establish the critical values which are used as decision thresholds to identify the regions with significant enriched concentrations. Several human genome regions are highly enriched in the occurrence of inverted repeats. This is observed in all the human chromosomes. The distribution of inverted repeat lengths varies along the genome. The majority of the regions with severely exaggerated enrichment contain mainly short length inverted repeats. There are also regions with regular peaks along the inverted repeats lengths distribution (periodic regularities) and other regions with exaggerated enrichment for long lengths (less frequent). However, adjacent regions tend to have similar distributions.

## 1 Introduction

The non-B DNA structures play important roles for biological processes (see for example, [1–6]). This work focus on the study of the lengths of potential hairpins/cruciforms non-B DNA structures. Various studies in the literature argue that cruciform structures are a common DNA feature important for regulating biological processes and that the genomes contain a remarkable number of inverted repeats in a non-random distribution (see [7] for a review).

Cruciform structures are formed by inverted repeats, which are composed by a single stranded sequence of nucleotides followed downstream by its reverse complement. There are several procedures and computational approaches to study inverted repeats and to describe the regional variation of inverted repeat lengths [8–10].

In this work we have developed a simulation scenario in order to highlight/identify the regions with exaggerated enrichment globally (of all lengths) and by lengths sections. We also present an exploratory analysis of all human chromosomes inverted repeat enrichment globally (in all genome) and in each chromosome separately.

## 2 Methods

This work uses the human genome (GRCh38 assembly sequences) and searches for features beyond the already well-known repetition structures published in the literature. Thus, the pre-masked sequences available from the UCSC Genome Browser webpage [11] with repeats reported by RepeatMasker [12] and Tandem Repeats Finder [13] masked with *N* symbols were used. The unknown or ambiguous nucleotides are usually coded with *N* symbols. All these ambiguous nucleotides are considered separators that split the sequence into a set of unambiguous subsequences.

Inverted repeats are nucleotide sequences that can form self complementary pairings between their two halves e.g. *CCTTACGnnnnnnCGTAAGG*, where {*A*, *C*, *G*, *T*} represent the DNA alphabet and *nnnnnn* represent a sequence of nucleotides with a known length.

In this work, we analyse the distribution of the distances between reverse complement sequences with *k* = 7 nucleotides, which is similar to the distribution of the lengths of the inverted repeats of 7 nucleotides. We study this distribution along the genome by dividing the complete genome in successive windows containing 10^5^ nucleotides.

For all words of length *k*, we compute the frequency distributions of each distance, *m*(*d*), between occurrences of each word and all succeeding reversed complements at distances between *k* and 4000.

### 2.1 Measuring the concentration of inverted repeats

In order to evaluate the behaviour of the observed values of the *m*(*d*), inverted repeat cumulative frequencies are compared to the corresponding expected values obtained from a Markov chain reference model of order 7.

#### 2.1.1 Expected values under higher order Markov chain for DNA sequences

Let *M*(*d*) be the random variable that represents the total number of inverted repeats occurrence at distance *d* in a genomic region of length *L* and *n*(*d*) the corresponding total number of possible word pairs at distance *d*, where *d* = *k*, *k* + 1, …, 4000 and *d* = *k* means that the two words (of length *k*) are in adjacent positions.

Let *p*(*d*) be the probability of occurrence of inverted repeats at distance *d*. If we assume the independence between trials, *M*(*d*) follows a binomial distribution, *M*(*d*) ⌢ *B*(*n*(*d*), *p*(*d*)) with expected value *n*(*d*)*p*(*d*) and standard deviation $\sqrt{n\left(d\right)p\left(d\right)\left(1-p\left(d\right)\right)}$ . We assumed a Markov model of order *k* to estimate *p*(*d*) since the occurrence of reversed complements cannot be considered independent.

We use a *z*-score, as proposed recently [14], as a measure between the observed values and the expected values obtained from the Markov model,

(1) $$Z\left(d\right)=\frac{M\left(d\right)-n\left(d\right)p\left(d\right)}{\sqrt{n\left(d\right)p\left(d\right)\left(1-p\left(d\right)\right)}}.$$

In order to measure the concentration of inverted repeats for a set of successive distances we compute the sum of all *T* values between two bounds (*d*1 and *d*2)

(2) $$S_{\left[d1,d2\right]}=\underset{d\in \left\{d1,\ldots ,d2\right\}}{\sum }T\left(d\right),$$

with *T*(*d*) a *z*-score adjusted to account for the effect of the presence of ambiguous symbols in the sequence [14].

#### 2.1.2 Simulation study

A control scenario simulation was developed and run to evaluate the results of the *S* measure under controlled conditions. Control scenario conditions:

– 24 sequences with the same size of each of the human chromosomes;
– the same number and in the same positions of the ambiguous symbols (*N*s) in human chromosomes;
– the DNA sequences were generated by a 7-order Markovian model;
– the probabilities of the words and the transition matrices of the Markov model, were estimated from each chromosome sequence.

We use the results of the simulation procedure to obtain a critical value for the *S* statistic (Equation (2)) under the assumption that the DNA sequences were generated by a 7-order Markovian model. An empirical distribution, of the *S* measure from the simulated sequences, was generated for each chromosome combining the contribution of all the windows in each chromosome.

We compute the critical values on the empirical distribution of the simulated genome, assuming a significance level of 5 %. The critical values are the 0.95 quantiles (cv) of the *S* values of all windows in each chromosome (or globally). Windows with *S* values surpassing the critical value are considered significantly enriched.

### 2.2 Data analysis

The data analysis is based on *M*(*d*) and *S*_[*d*1,*d*2]_. It is divided into 3 parts:

– comparison of inverted repeats enrichment between chromosomes;
– analysis of inverted repeats enrichment as a function of inverted repeats length;
– analysis of the inverted repeats enrichment as a function of position along each chromosome.

Inverted repeat lengths were grouped into nine classes: *I*_*t*_ = *S*_[7,4000]_, *I*_1_ = *S*_[7,500]_, *I*_2_ = *S*_[501,1000]_, *I*_3_ = *S*_[1001,1500]_, *I*_4_ = *S*_[1501,2000]_, *I*_5_ = *S*_[2001,2500]_, *I*_6_ = *S*_[2501,3000]_, *I*_7_ = *S*_[3001,3500]_, *I*_8_ = *S*_[3501,4000]_.

## 3 Results and discussion

### 3.1 Comparison of inverted repeats enrichment between chromosomes

**Figure 1:**
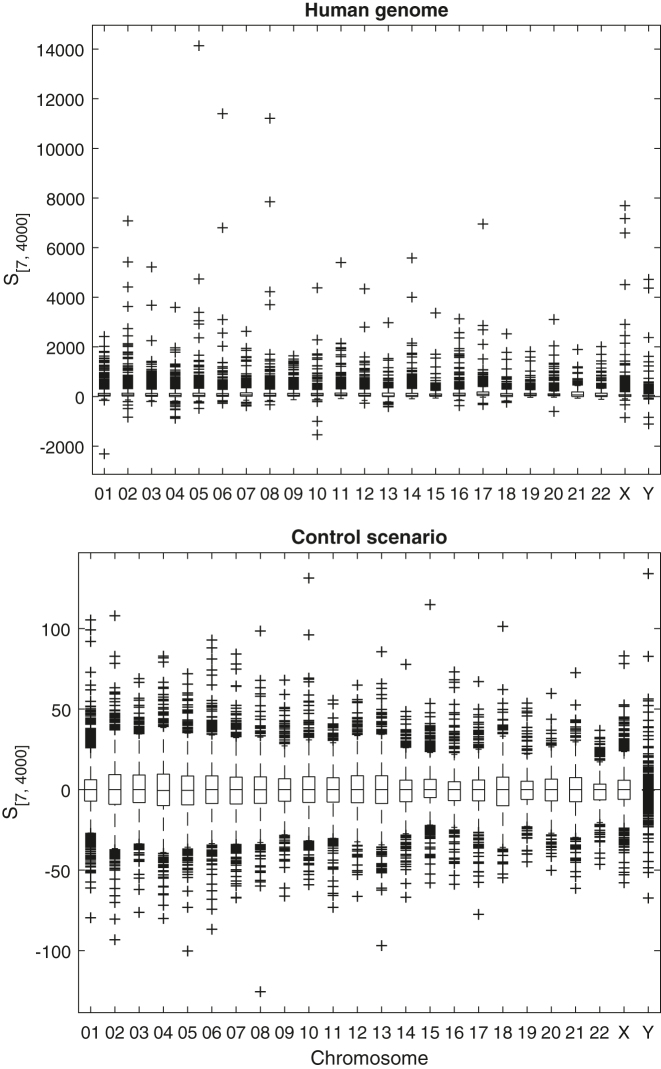
Boxplot of *S*_[7,4000]_ values for each chromosome: top, human genome; bottom, control scenario.

Figure 1 shows the boxplots of *S*_[7,4000]_ for all chromosomes and all considered inverted repeat lengths (length class *I*_*t*_), both for the human genome and the control scenario. The distributions of the *S* values in the human genome are reasonably similar for the various chromosomes: all distributions show positive mean, positive skew and similar dispersion. The distributions of the control scenario are clearly different from those of the human genome: all distributions have null mean, are symmetric and have a comparatively much lower dispersion.

Table 1 shows the percentage of windows that were considered to have significantly enriched concentration of inverted repeats in each chromosome and for each inverted repeat length class. The percentage of enriched windows for all considered inverted repeats lengths (class *I*_*t*_) in the complete genome is quite large (66.3 %). This confirms the strong positive skew of the *S* distribution previously observed.

**Table 1:** Percentage of windows with significantly enriched concentration of inverted repeats for each length class. Column “wins” shows the total number of windows in each chromosome. Row “Global %” shows the percentages for the complete genome. Row “Mean cv” shows the weighted mean of the critical values.

Chr	Wins	*I*_*t*_	*I*_1_	*I*_2_	*I*_3_	*I*_4_	*I*_5_	*I*_6_	*I*_7_	*I*_8_
1	2490	70.6	75.4	45.9	31.0	23.8	15.9	12.2	9.8	8.8
2	2422	67.7	72.9	44.2	30.0	19.2	13.7	11.5	9.0	7.0
3	1983	61.9	67.3	40.0	27.8	20.1	14.3	11.0	8.3	7.4
4	1903	60.4	64.1	41.3	25.3	19.3	11.0	8.8	6.5	5.6
5	1816	59.9	65.5	42.2	29.6	20.6	11.8	9.3	7.2	6.7
6	1709	60.8	68.5	38.2	27.5	18.4	13.6	10.6	8.4	6.7
7	1594	68.3	73.4	46.0	33.6	21.8	16.4	13.2	9.3	7.5
8	1452	67.6	72.2	42.4	29.8	19.8	14.3	11.0	9.6	6.3
9	1384	71.6	73.9	42.6	30.8	21.2	14.9	10.6	8.5	8.1
10	1338	78.7	82.7	44.3	30.2	19.1	12.8	8.8	8.1	7.5
11	1351	79.8	82.9	51.2	33.0	22.6	16.4	14.1	10.9	8.6
12	1333	66.1	71.1	42.6	31.7	20.0	14.1	12.0	8.9	7.4
13	1144	48.9	51.8	36.5	28.1	20.3	12.0	9.3	8.0	5.7
14	1071	61.7	66.3	42.7	29.8	22.2	16.4	11.8	9.1	9.0
15	1020	65.0	69.8	37.1	26.3	17.2	15.6	11.5	10.2	7.5
16	904	78.9	80.3	42.1	31.4	23.7	18.7	14.8	11.3	10.6
17	833	85.7	89.3	59.4	40.6	30.3	23.9	18.4	14.6	11.0
18	804	60.2	64.7	36.9	25.1	17.8	11.6	9.3	6.1	6.7
19	587	83.3	84.7	52.8	40.7	27.6	22.8	18.1	14.8	10.9
20	645	85.0	86.5	46.4	28.1	16.1	16.6	9.6	8.4	6.7
21	468	62.2	65.6	51.3	40.8	22.6	15.4	13.5	11.1	10.7
22	509	62.5	63.5	40.3	34.6	23.8	18.3	16.3	13.2	10.8
*X*	1561	56.6	60.8	31.3	20.2	14.0	9.8	7.5	5.6	6.1
*Y*	573	33.2	34.0	23.7	17.8	10.8	8.9	6.3	6.1	3.5
Global %		66.3	70.6	42.4	29.6	20.4	14.5	11.3	8.9	7.5
Mean cv		23.8	18.9	8.0	3.8	1.9	1.1	0.6	0.3	0.1

### 3.2 Analysis of inverted repeats enrichment as a function of inverted repeats length

Table 1 also shows the percentage of windows with significantly enriched concentration of inverted repeats in each chromosome, for each length class (*I*_1_ through *I*_8_). The percentage decreases with the increase of inverted repeat length.

**Figure 2:**
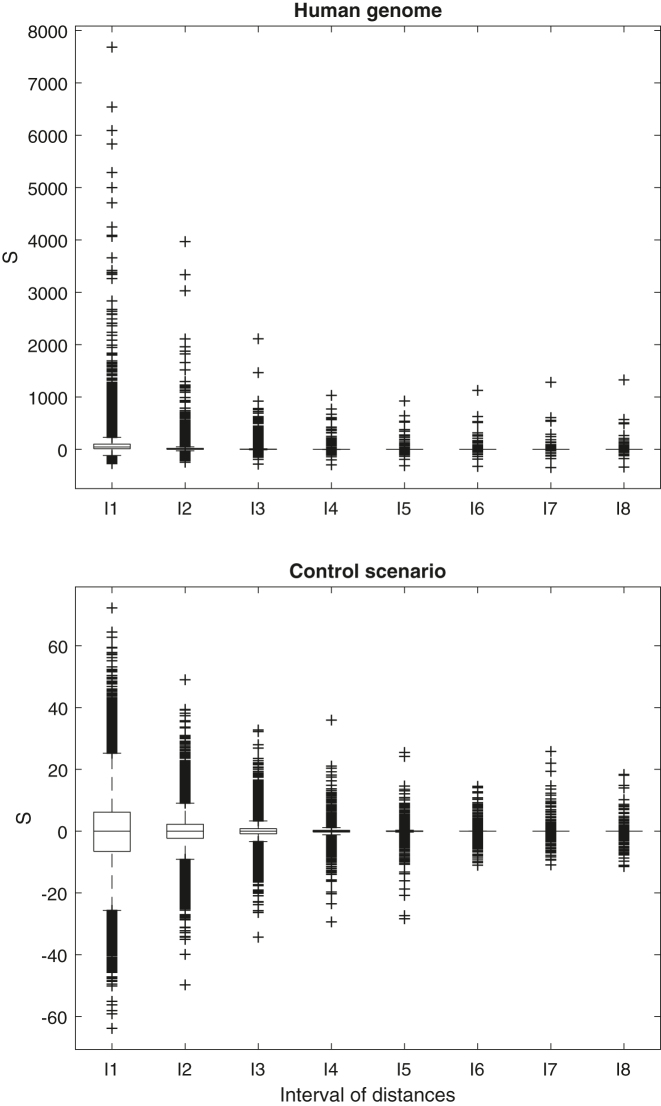
Boxplot of *S*_[*d*1,*d*2]_ values for 8 length classes: top, human genome; bottom, control scenario.

Figure 2 shows the boxplots of *S* values for each length class in the human genome and in the control scenario.

The human genome reveals some regions with very high and significant enrichment in all length classes. The control scenario shows that the dispersion of *S* decreases with the increase of inverted repeats length. This reveals that the *S* measure is sensitive to the inverted repeat length. This limitation of the measure does not compromise our analysis, since critical values were obtained from the control scenario in each length class.

**Figure 3:**
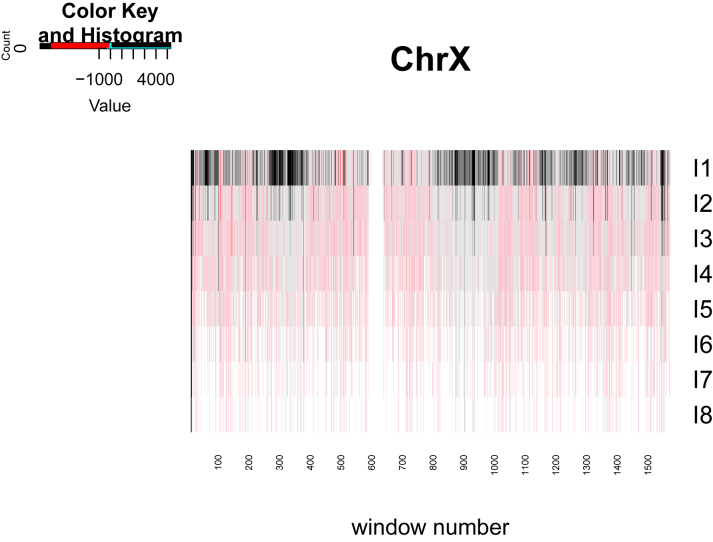
Heatmap of the *S* values for the inverted repeats length classes *I*_1_, *I*_2_, …, *I*_8_ in chromosome X. Black shows enrichment and red shows reduction of the frequency of inverted repeats.

Figure 3 shows the variations of *S* in the different length classes along chromosome X.

Windows with similar enrichment seem to form regional clusters along the genome.

### 3.3 Analysis of the inverted repeats enrichment as a function of position along each chromosome

**Figure 4:**
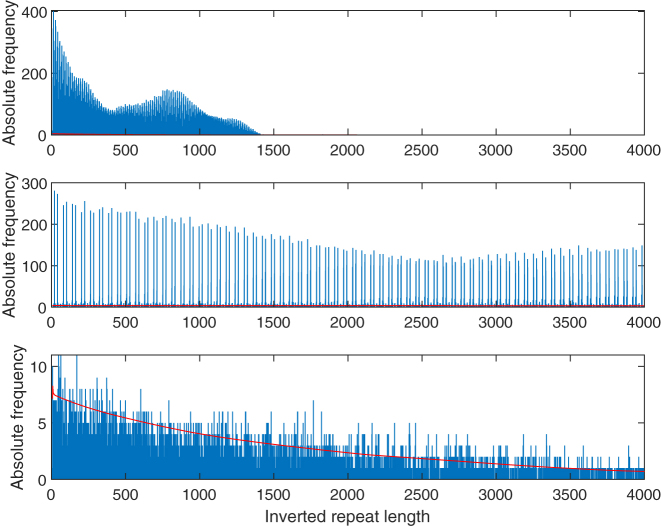
Inverted repeat length frequencies of three windows in chromosome X: top, window with the highest *S*_[7,4000]_, chrX:53000001:53100000; middle, window with the highest *S*_[2001,2500]_, chrX:100001:200000; bottom, window with the lowest *S*_[7,4000]_ chrX:36700001:36800000. The red solid line represents the expected value for the absolute frequency.

Figure 4 shows the absolute frequencies of occurrence of each inverted repeat length in three different windows of chromosome X. The top/bottom plots pertain to the windows with the highest/lowest *S*_[7,4000]_ values in that chromosome. The middle plot pertains to the window with the highest *S*_[2001,2500]_ values.

The Supplementary Material contains the absolute frequency plots for windows selected according to the same criteria in every chromosome.

The selected windows display very different distributions. The distributions on the top and middle plots both present frequency values much higher than the expected values. The bottom plot represents a rare example of a window with negative *S* values. The periodic regularities seen in the middle plot were previously identified and studied in [9].

## 4 Conclusions

The analysis carried out in this work revealed several human genome regions with highly enriched occurrence of inverted repeats in all human chromosomes. The enrichment in inverted repeats concentration is not uniform along the genome and it depends on the repeats lengths, being more prominent for short lengths.

Even though we removed well known repetitive sequences, we still found regions with atypically enriched concentration of inverted repeats. Further studies for understanding the reasons for this phenomenon are needed, which may imply analysing the genomic word composition of these regions.

## Supplementary Material

Supplementary Material Details
