# Supplementary material for: Concentration of inverted repeats along human DNA
Source: J Integr Bioinform. 2023 Jul 25;20(2):20220052. doi: 10.1515/jib-2022-0052 (PMC10561070; doi:10.1515/jib-2022-0052)
Supplement: Supplementary file 1 — Supplementary Material Details [file j_jib-2022-0052_suppl_001.zip › Readme.pdf]

Supplementary Material for paper
"Concentration of Inverted Repeats along human DNA"

Carlos A.C. Bastos, Vera Afreixo, João M.O.S. Rodrigues, Armando J. Pinho

Each provided file, `fig_dists_chr#.pdf`, shows the inverted repeat length frequencies of three windows for the chromosome chr#:

- Top plot displays the distribution for the window with the highest $S_{[7,4000]}$.
- Middle plot displays the distribution for the window with the highest $S_{[2001,2500]}$.
- Bottom plot displays the distribution for the window with the lowest $S_{[7,4000]}$.

The position of the selected window is included in the title of each plot.

The red solid line represents the expected value for the absolute frequency.

File `fig_dists_chrX.pdf` corresponds to Figure 4 in section 3.3 of the paper
