# Supplementary figures and images for: Concentration of inverted repeats along human DNA
Source: J Integr Bioinform. 2023 Jul 25;20(2):20220052. doi: 10.1515/jib-2022-0052 (PMC10561070; doi:10.1515/jib-2022-0052)

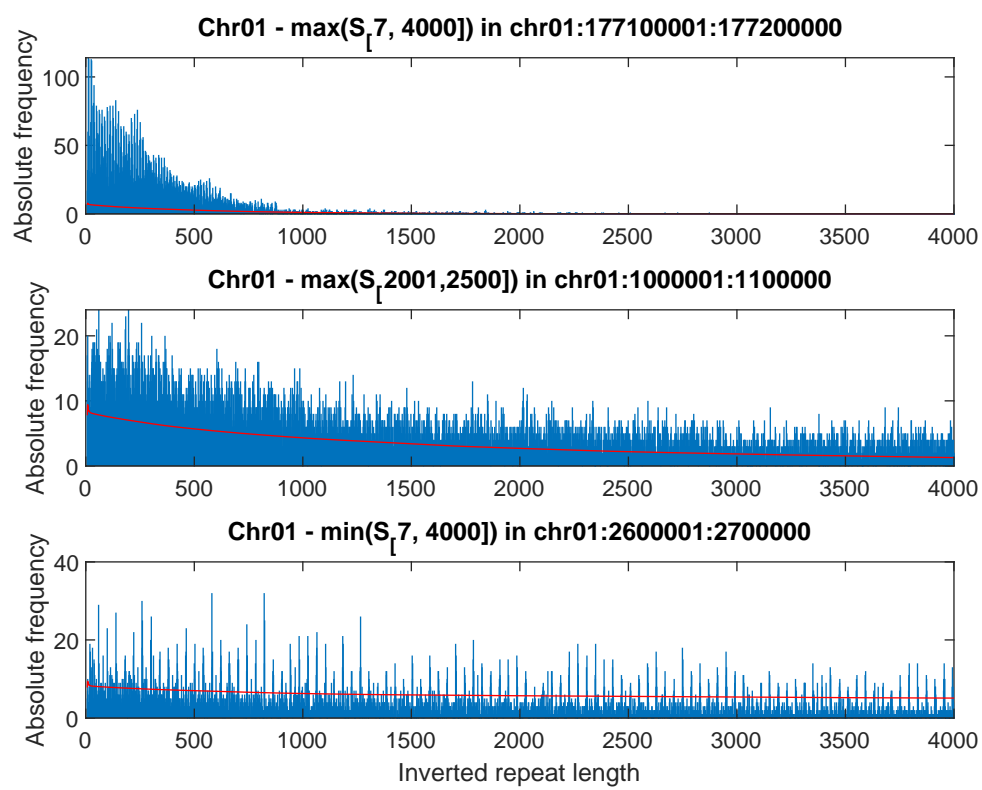

Supplement: Supplementary file 1 — Supplementary Material Details [file j_jib-2022-0052_suppl_001.zip › fig_dists_chr01.pdf]

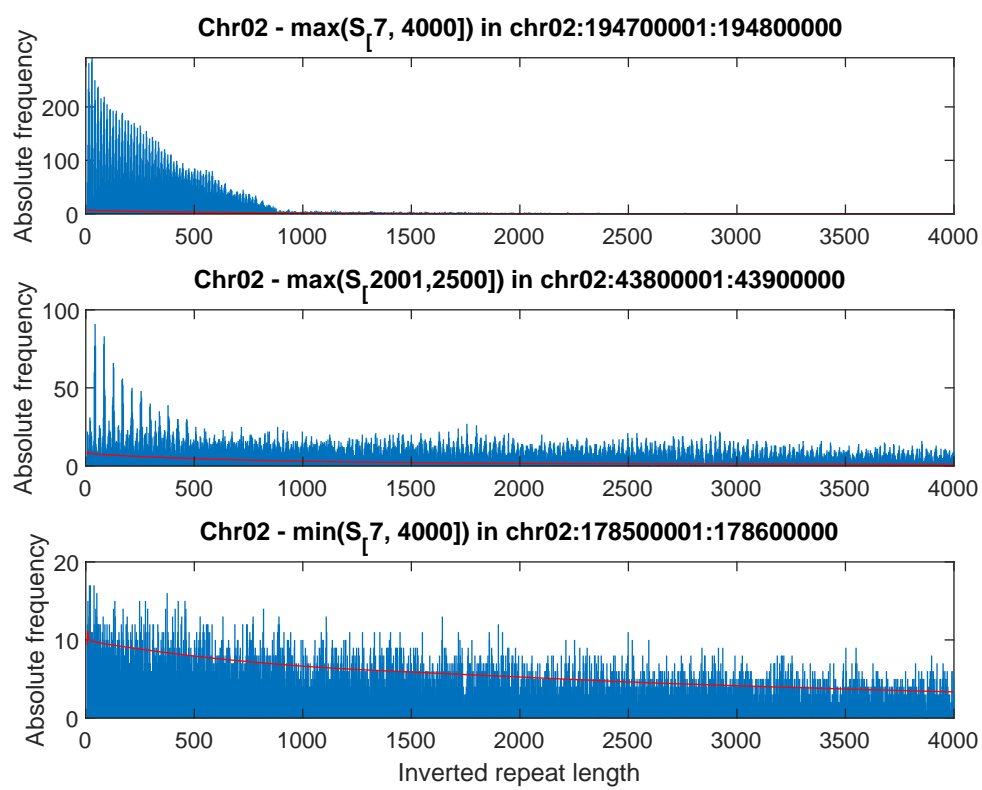

Supplement: Supplementary file 1 — Supplementary Material Details [file j_jib-2022-0052_suppl_001.zip › fig_dists_chr02.pdf]

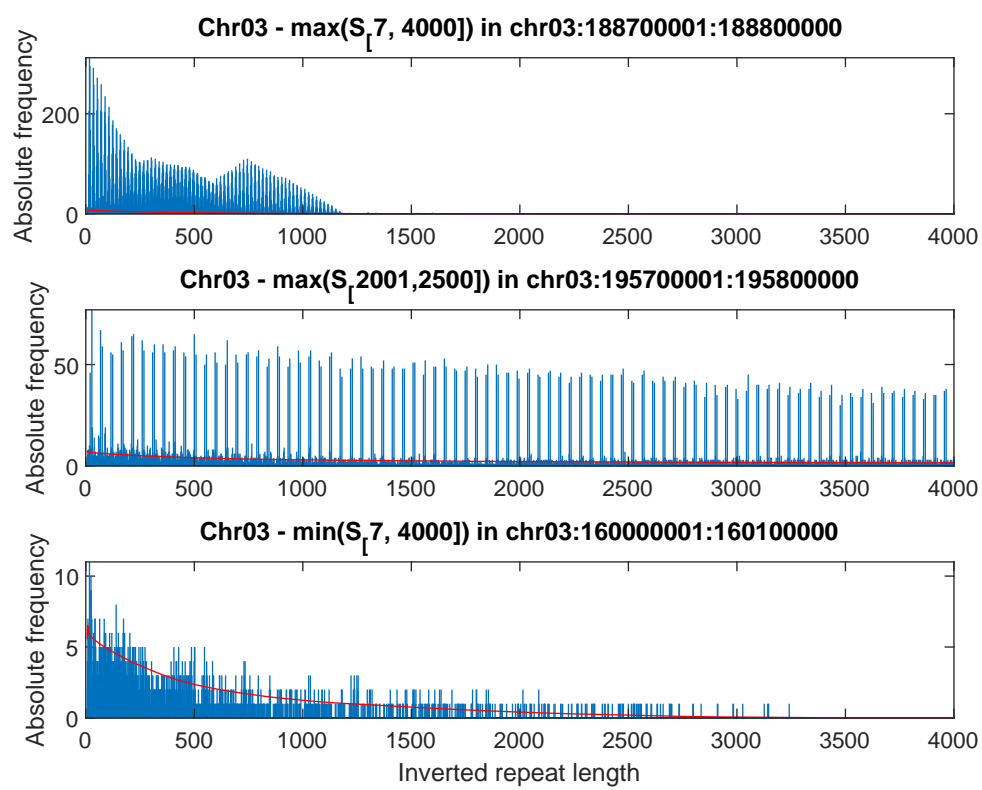

Supplement: Supplementary file 1 — Supplementary Material Details [file j_jib-2022-0052_suppl_001.zip › fig_dists_chr03.pdf]

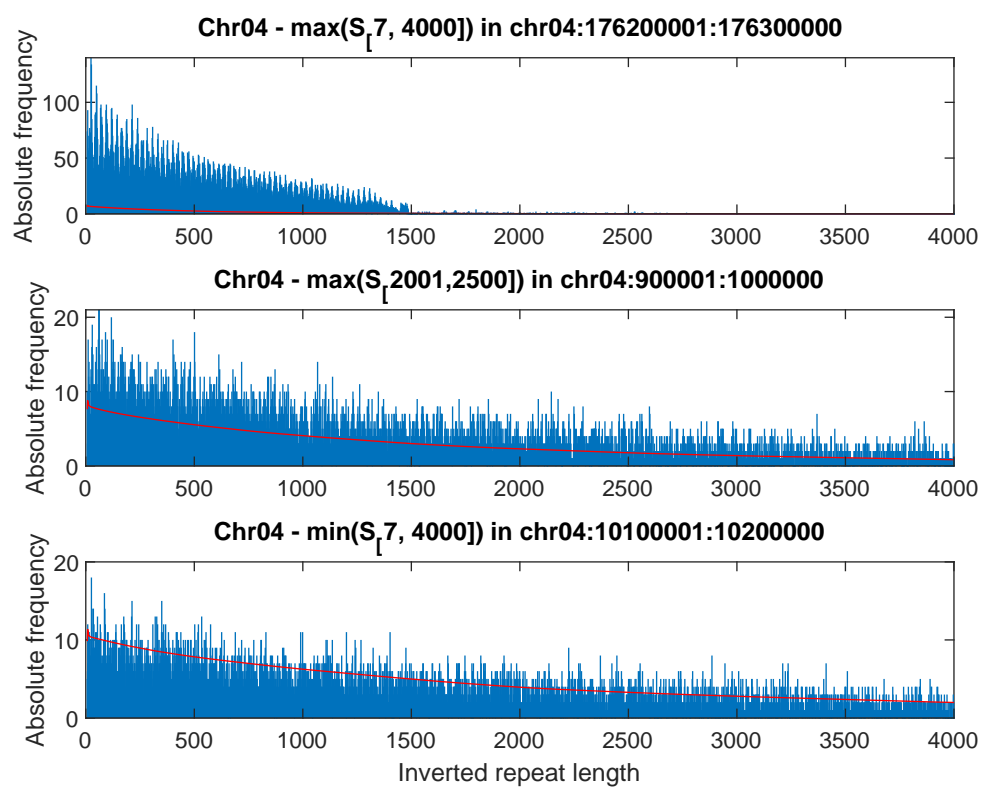

Supplement: Supplementary file 1 — Supplementary Material Details [file j_jib-2022-0052_suppl_001.zip › fig_dists_chr04.pdf]

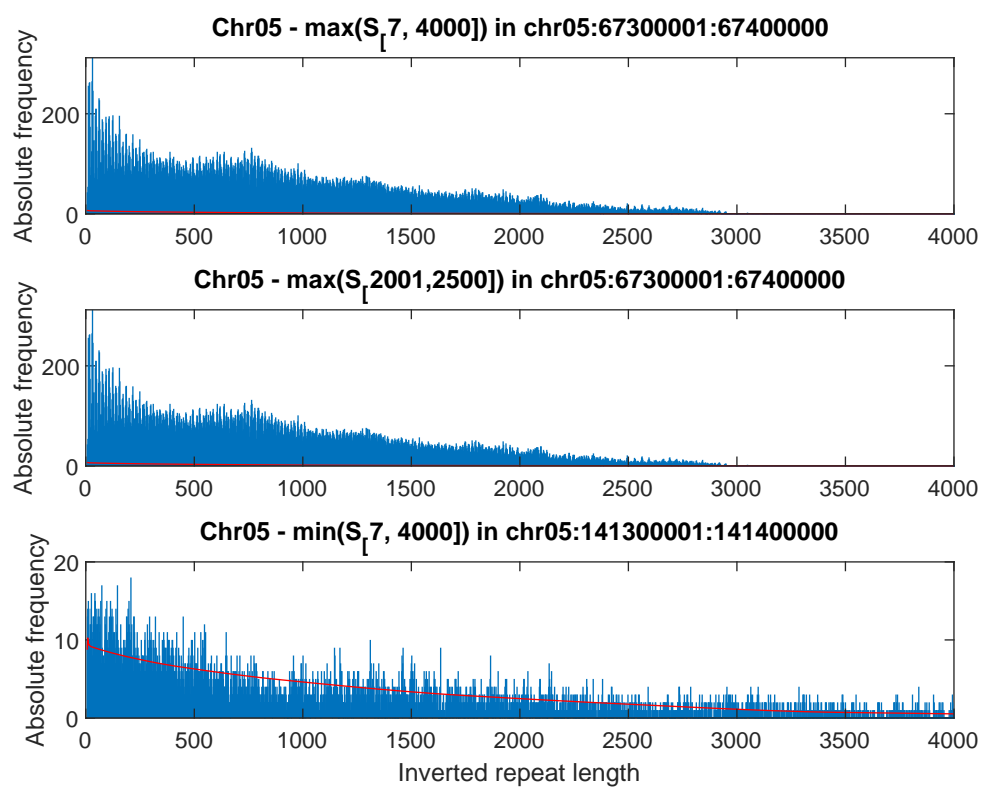

Supplement: Supplementary file 1 — Supplementary Material Details [file j_jib-2022-0052_suppl_001.zip › fig_dists_chr05.pdf]

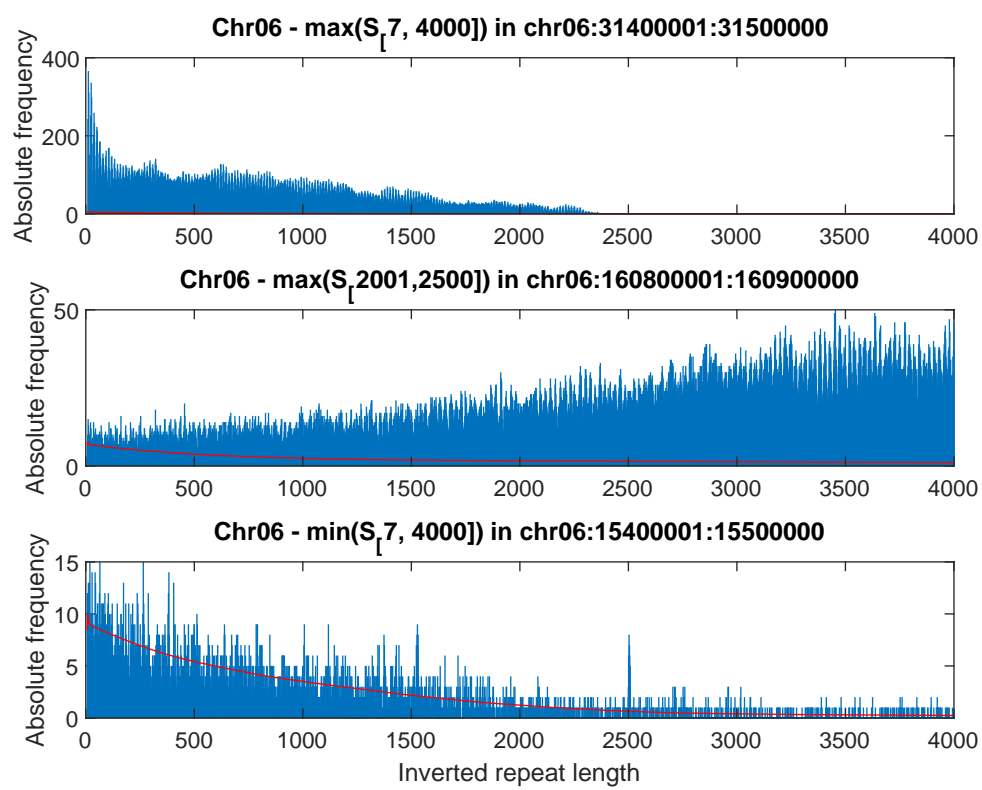

Supplement: Supplementary file 1 — Supplementary Material Details [file j_jib-2022-0052_suppl_001.zip › fig_dists_chr06.pdf]

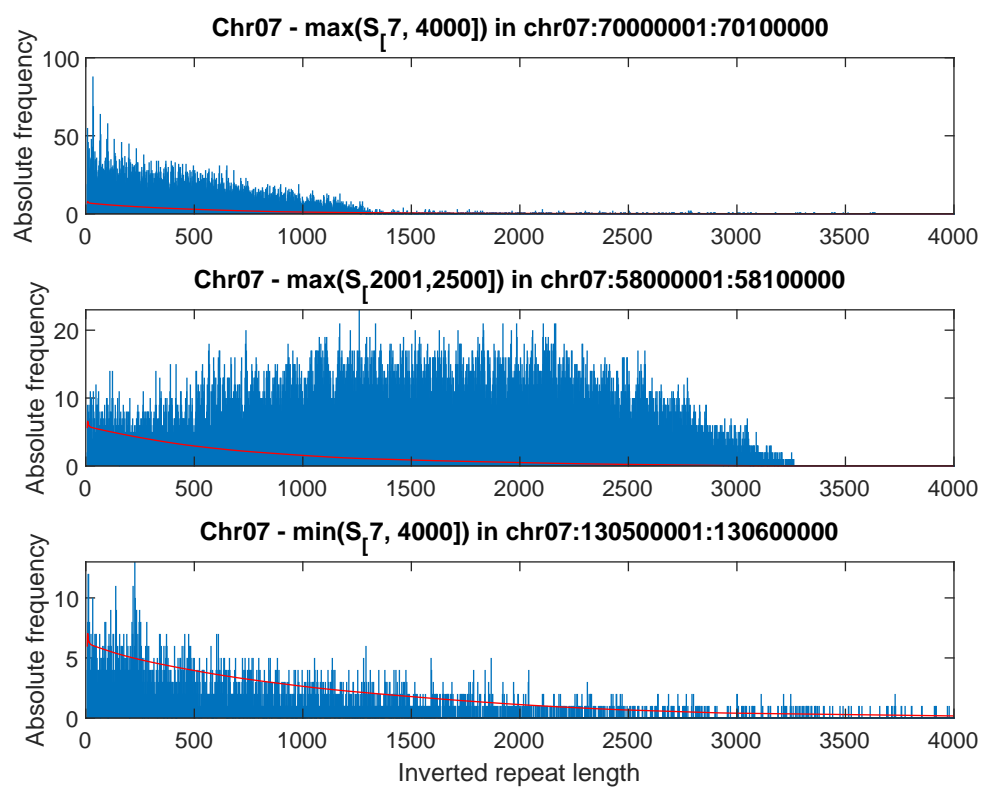

Supplement: Supplementary file 1 — Supplementary Material Details [file j_jib-2022-0052_suppl_001.zip › fig_dists_chr07.pdf]

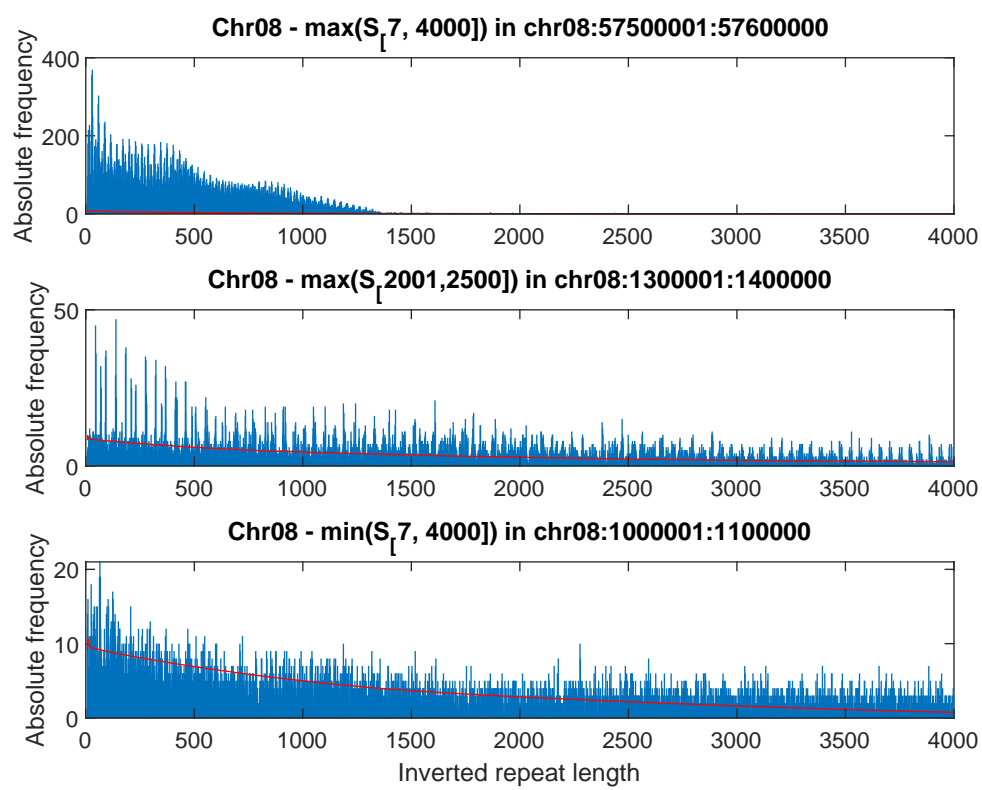

Supplement: Supplementary file 1 — Supplementary Material Details [file j_jib-2022-0052_suppl_001.zip › fig_dists_chr08.pdf]

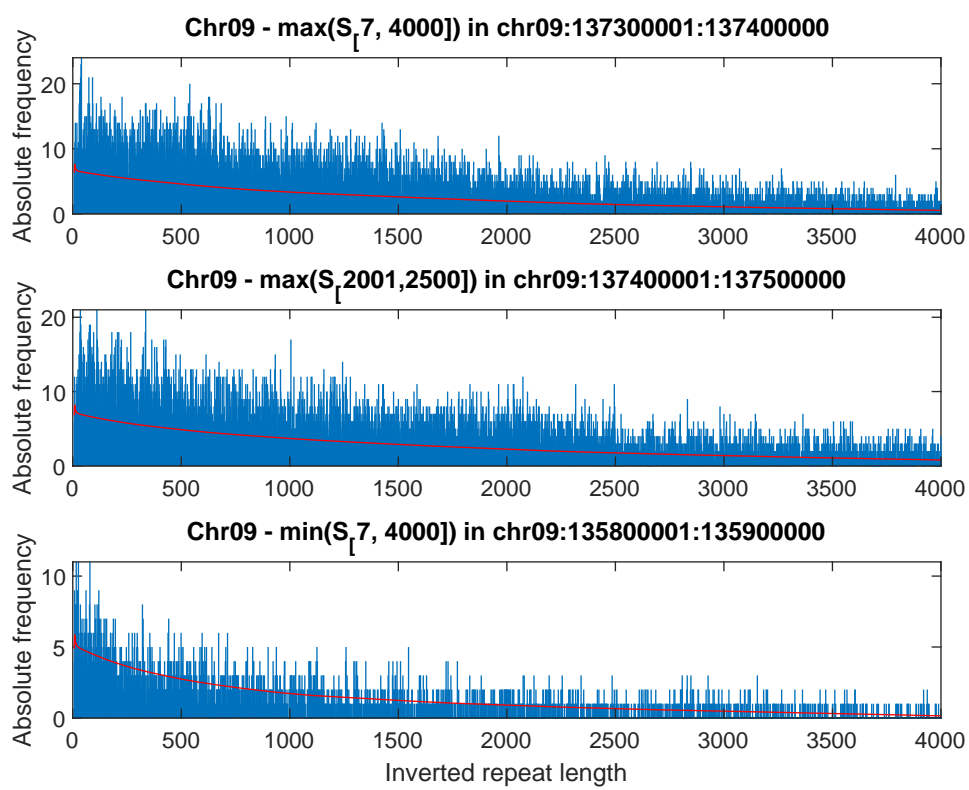

Supplement: Supplementary file 1 — Supplementary Material Details [file j_jib-2022-0052_suppl_001.zip › fig_dists_chr09.pdf]

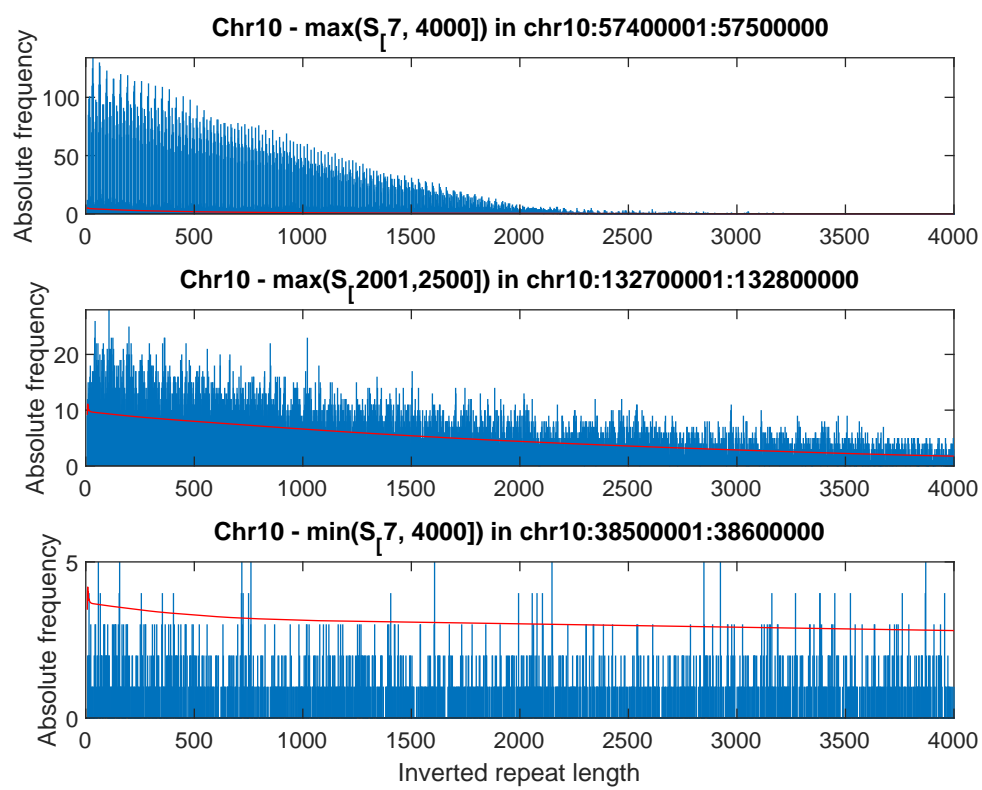

Supplement: Supplementary file 1 — Supplementary Material Details [file j_jib-2022-0052_suppl_001.zip › fig_dists_chr10.pdf]

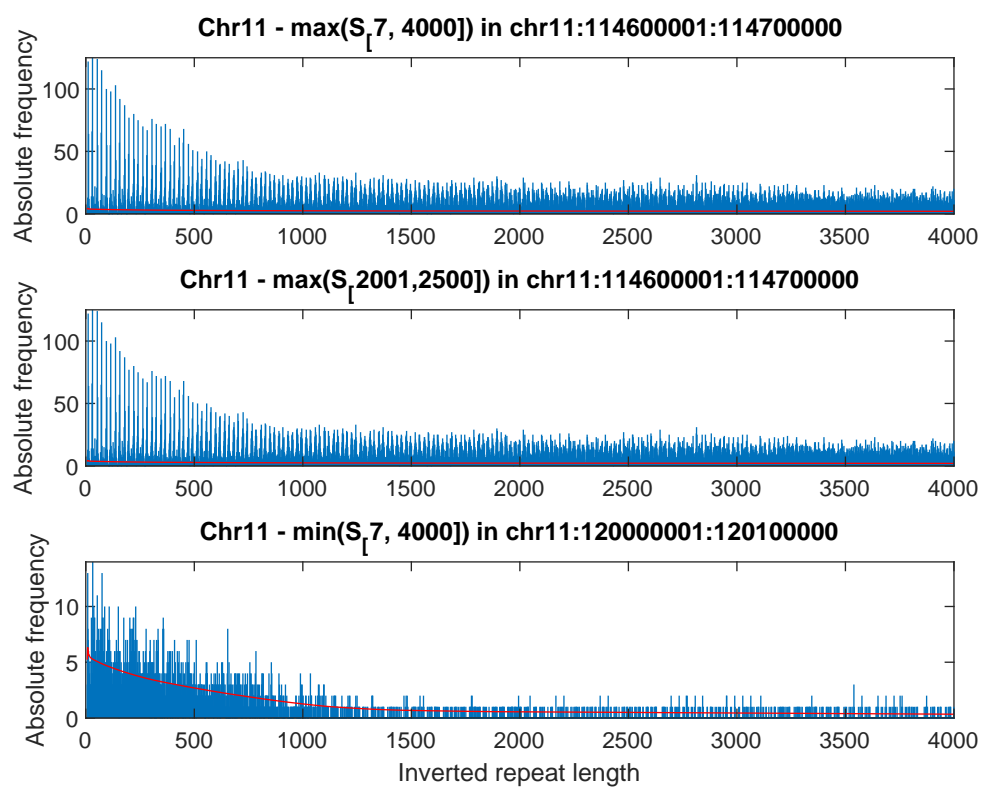

Supplement: Supplementary file 1 — Supplementary Material Details [file j_jib-2022-0052_suppl_001.zip › fig_dists_chr11.pdf]

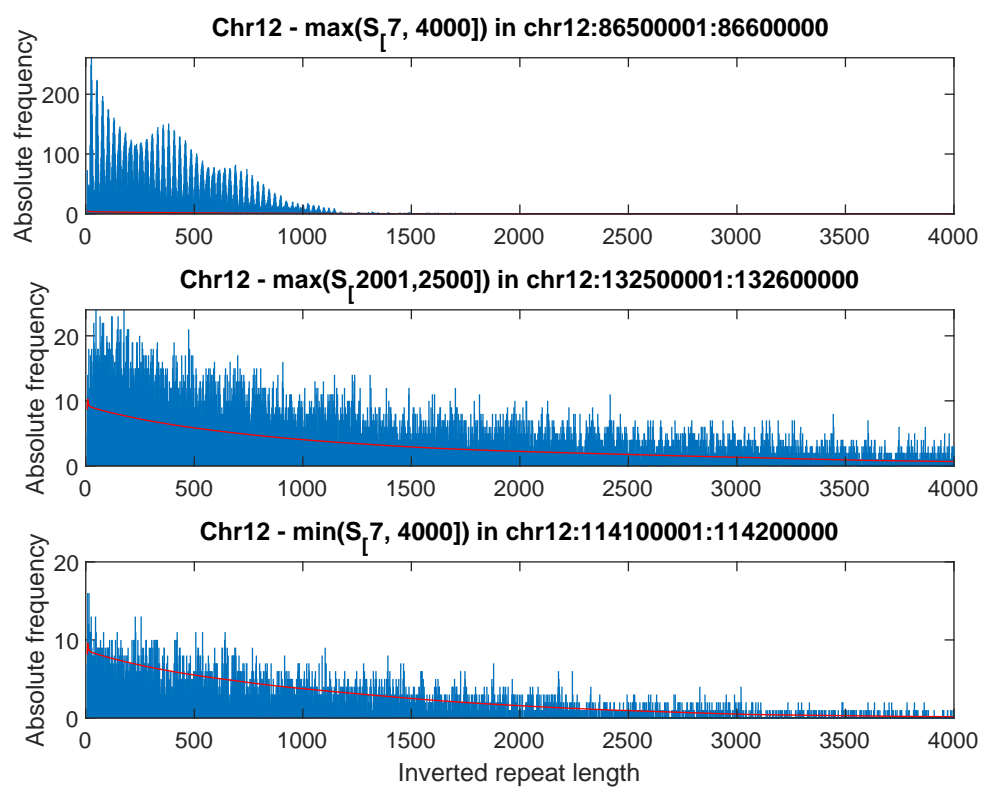

Supplement: Supplementary file 1 — Supplementary Material Details [file j_jib-2022-0052_suppl_001.zip › fig_dists_chr12.pdf]

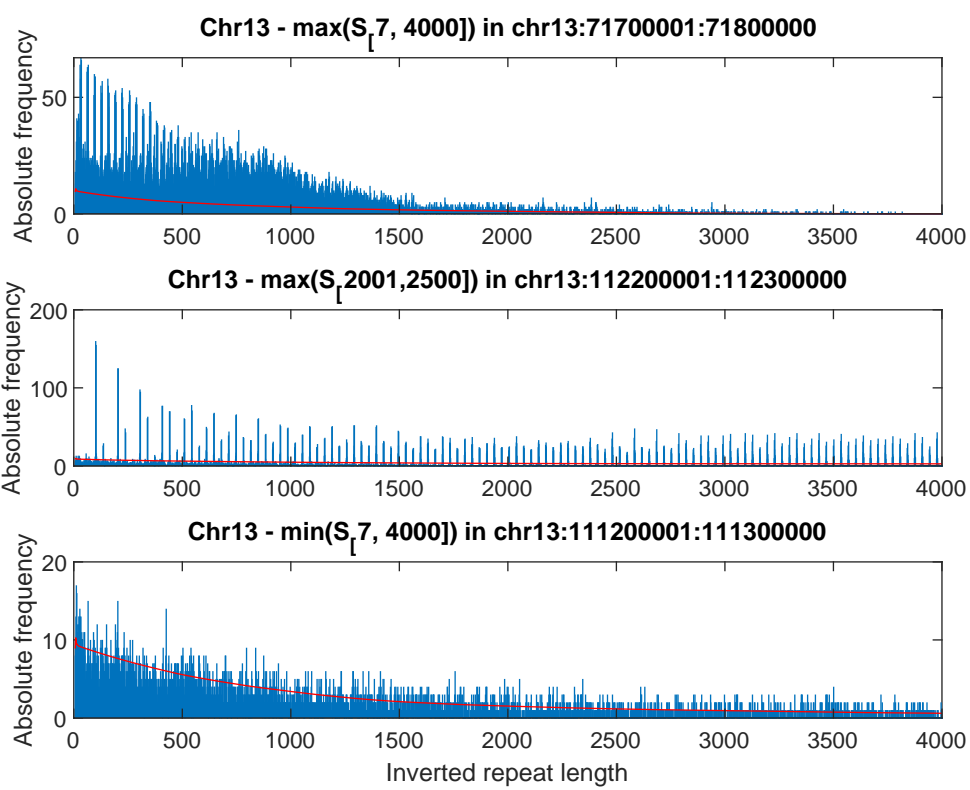

Supplement: Supplementary file 1 — Supplementary Material Details [file j_jib-2022-0052_suppl_001.zip › fig_dists_chr13.pdf]

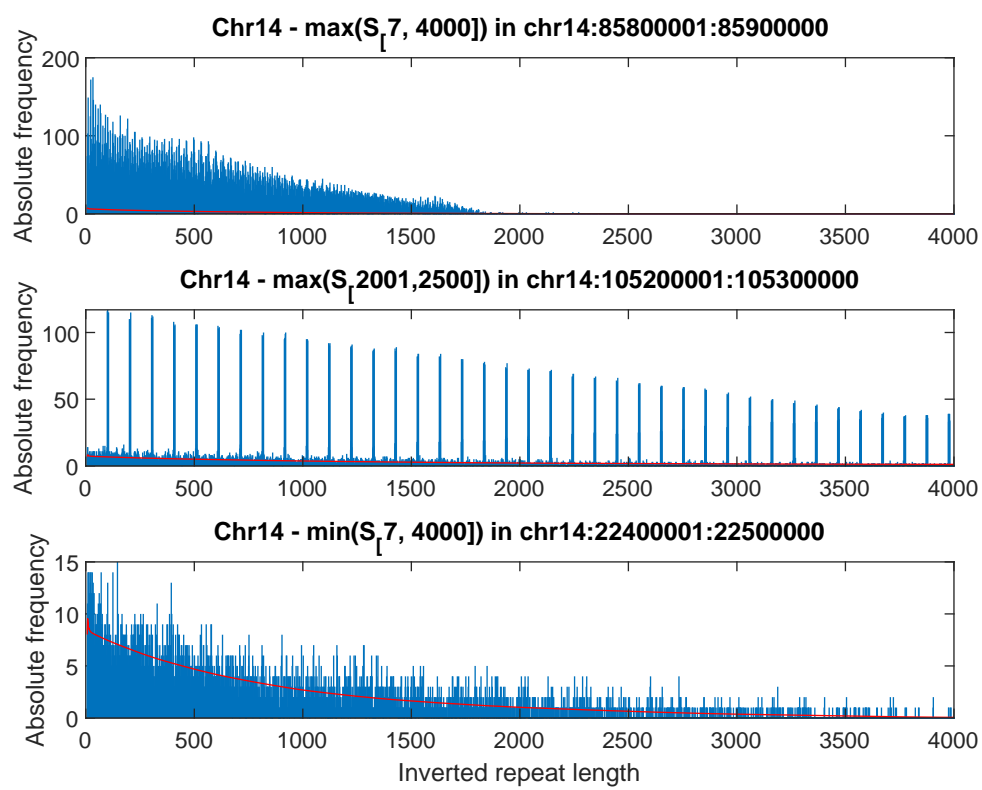

Supplement: Supplementary file 1 — Supplementary Material Details [file j_jib-2022-0052_suppl_001.zip › fig_dists_chr14.pdf]

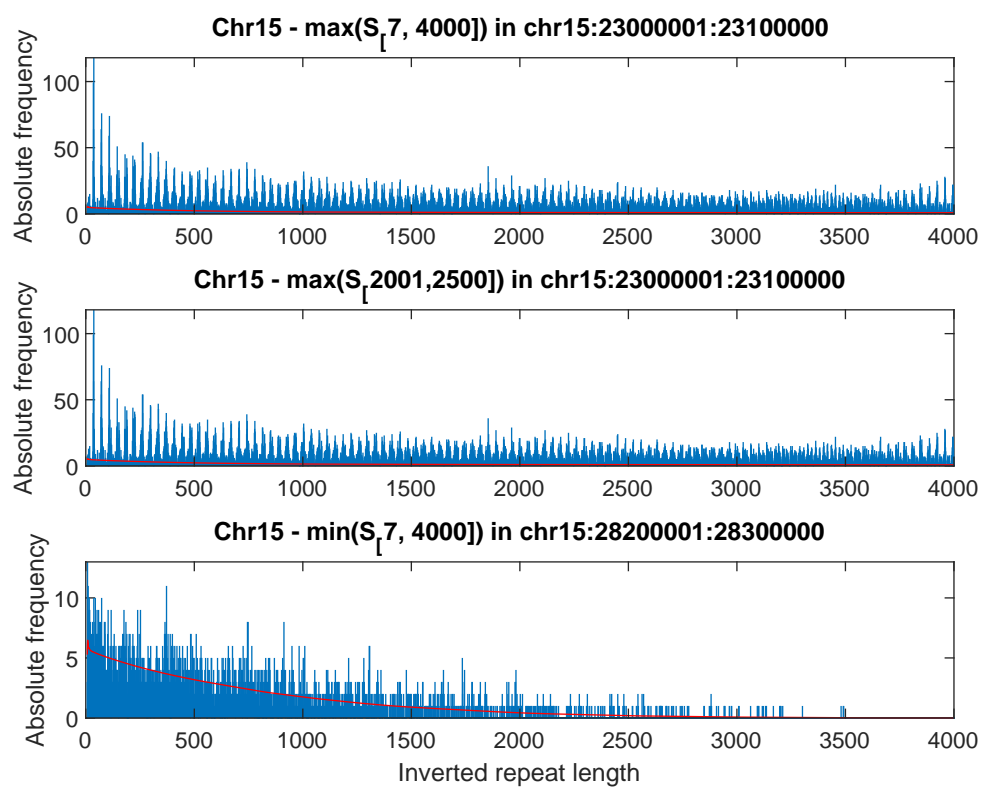

Supplement: Supplementary file 1 — Supplementary Material Details [file j_jib-2022-0052_suppl_001.zip › fig_dists_chr15.pdf]

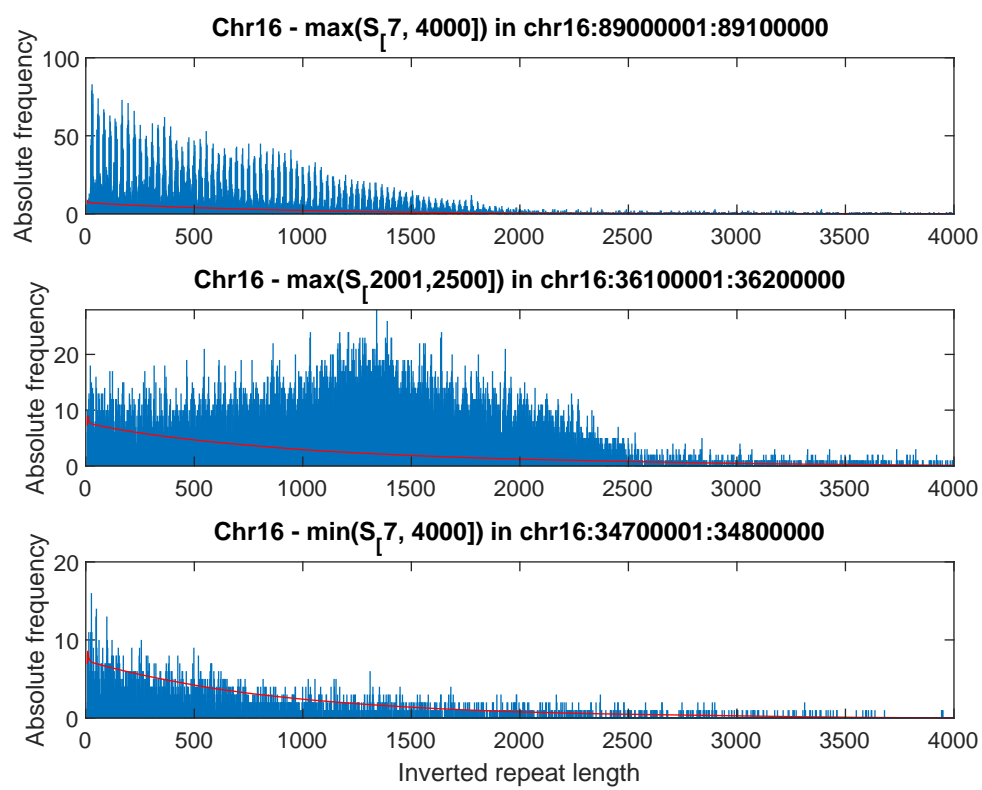

Supplement: Supplementary file 1 — Supplementary Material Details [file j_jib-2022-0052_suppl_001.zip › fig_dists_chr16.pdf]

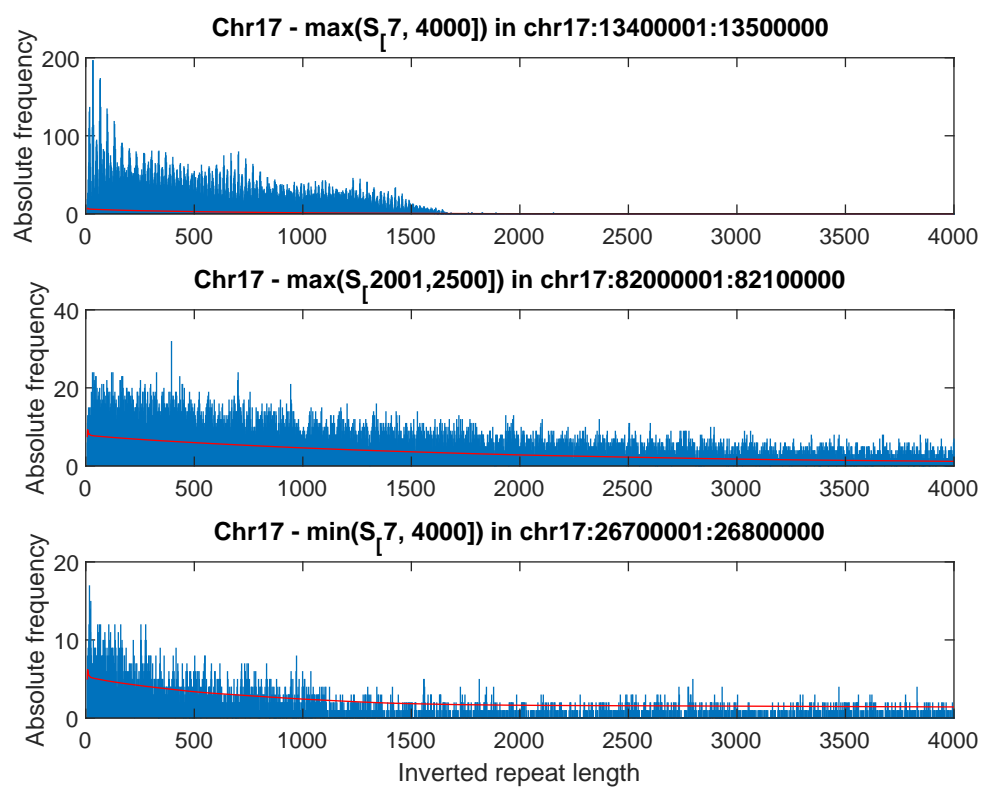

Supplement: Supplementary file 1 — Supplementary Material Details [file j_jib-2022-0052_suppl_001.zip › fig_dists_chr17.pdf]

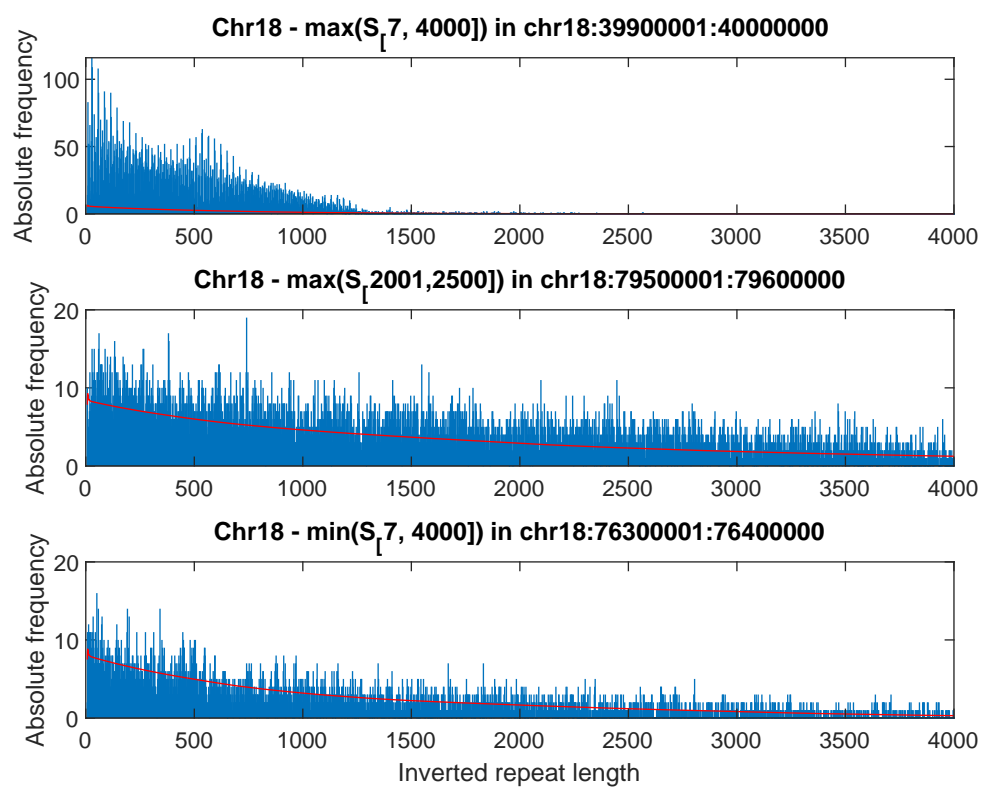

Supplement: Supplementary file 1 — Supplementary Material Details [file j_jib-2022-0052_suppl_001.zip › fig_dists_chr18.pdf]

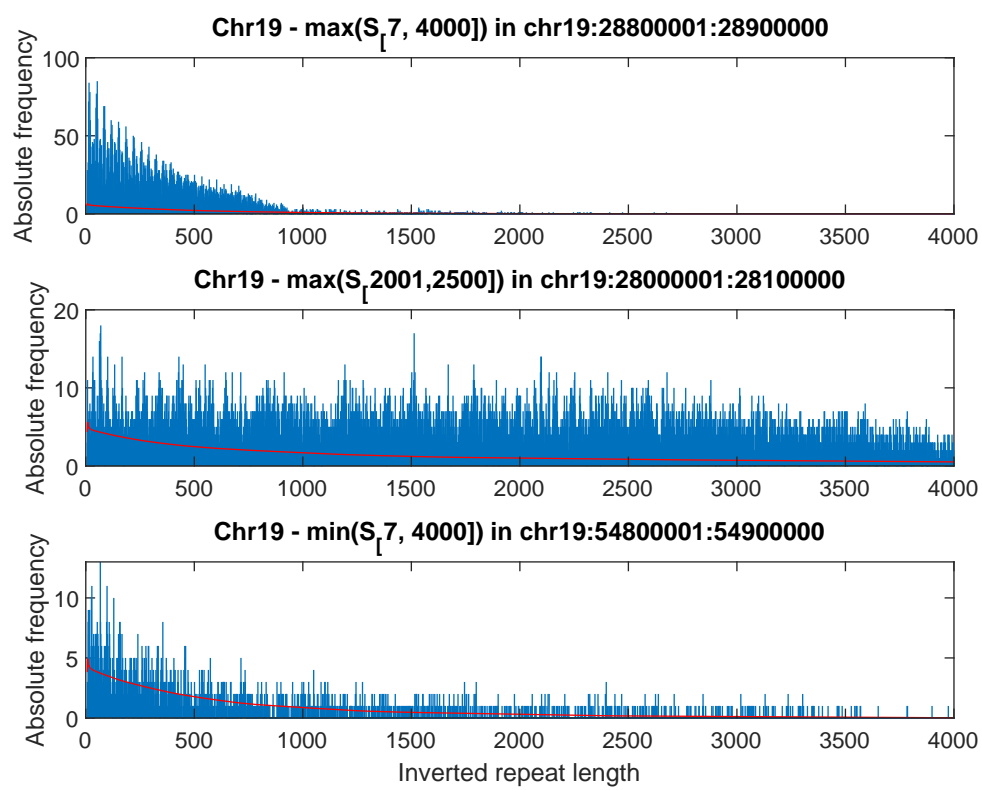

Supplement: Supplementary file 1 — Supplementary Material Details [file j_jib-2022-0052_suppl_001.zip › fig_dists_chr19.pdf]

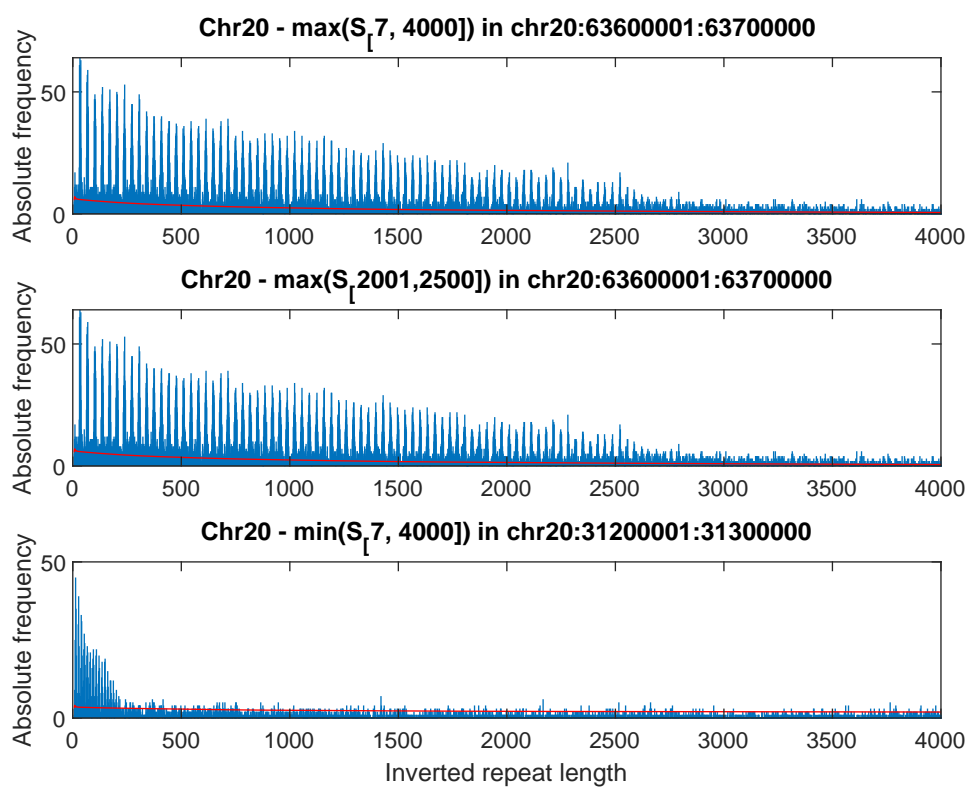

Supplement: Supplementary file 1 — Supplementary Material Details [file j_jib-2022-0052_suppl_001.zip › fig_dists_chr20.pdf]

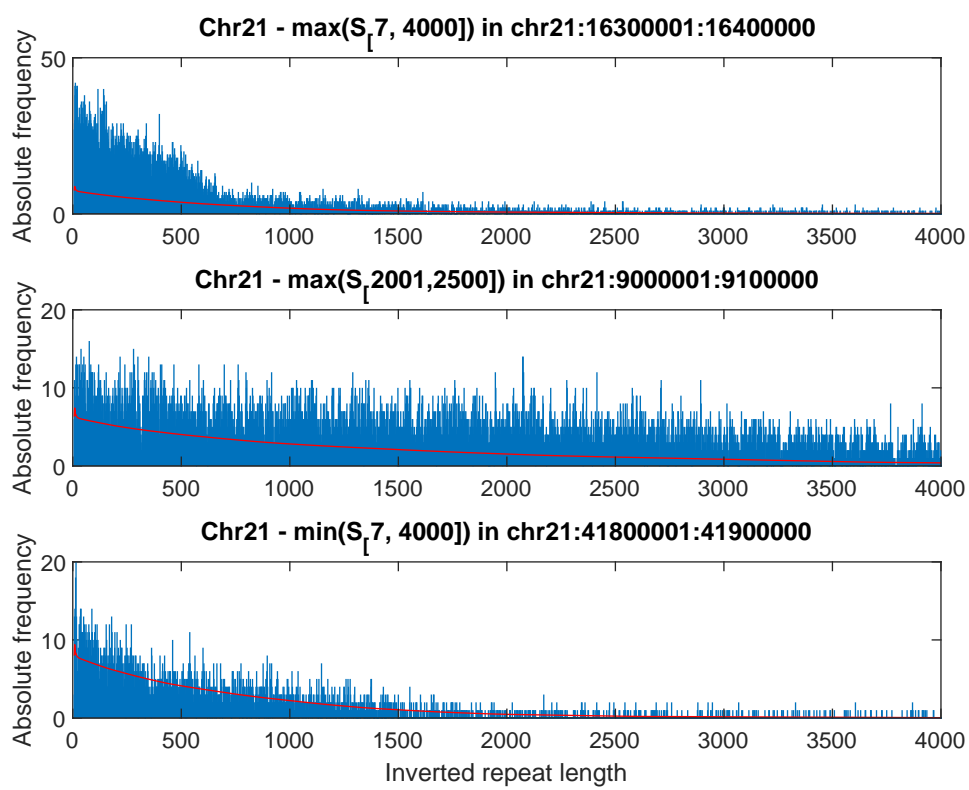

Supplement: Supplementary file 1 — Supplementary Material Details [file j_jib-2022-0052_suppl_001.zip › fig_dists_chr21.pdf]

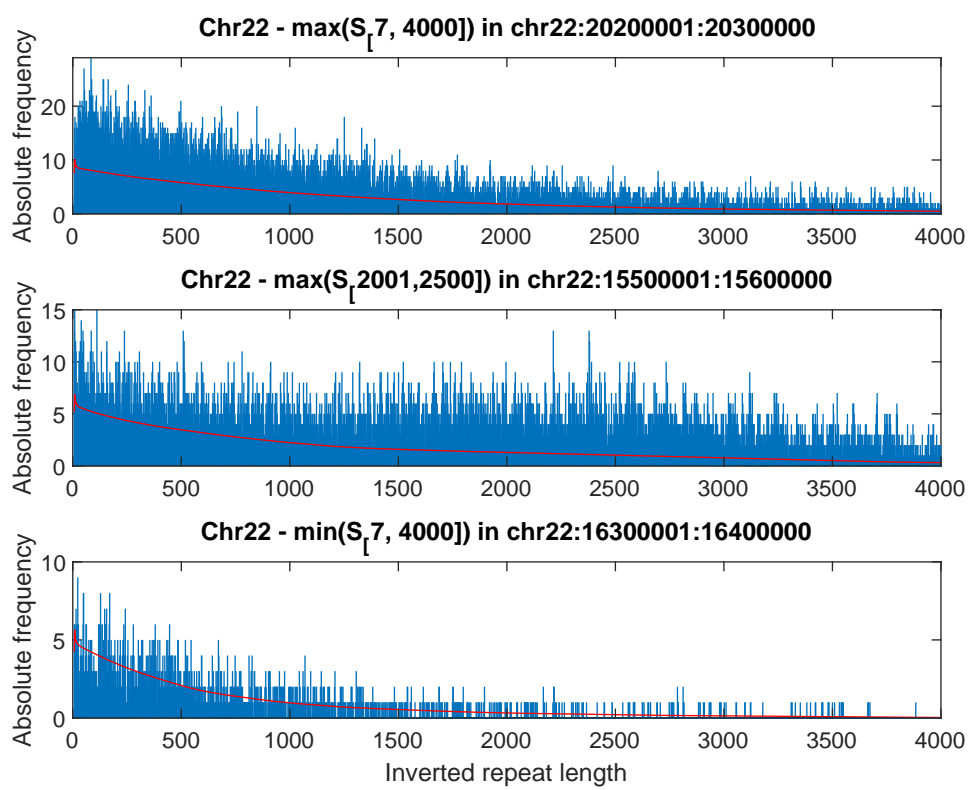

Supplement: Supplementary file 1 — Supplementary Material Details [file j_jib-2022-0052_suppl_001.zip › fig_dists_chr22.pdf]

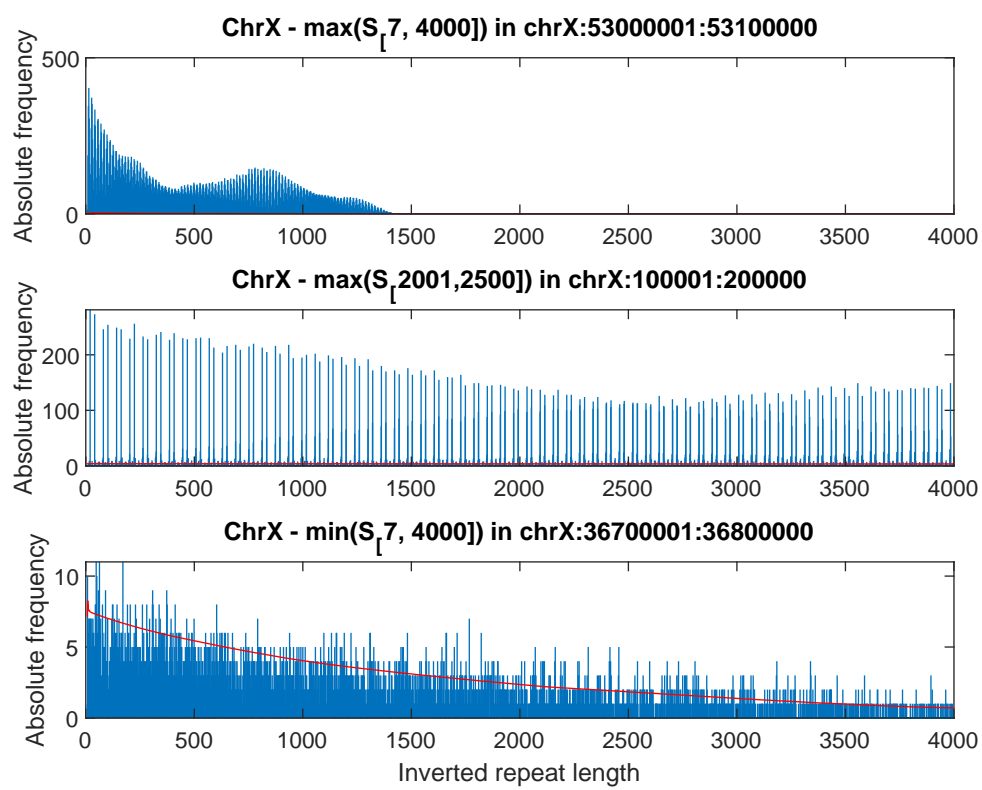

Supplement: Supplementary file 1 — Supplementary Material Details [file j_jib-2022-0052_suppl_001.zip › fig_dists_chrX.pdf]

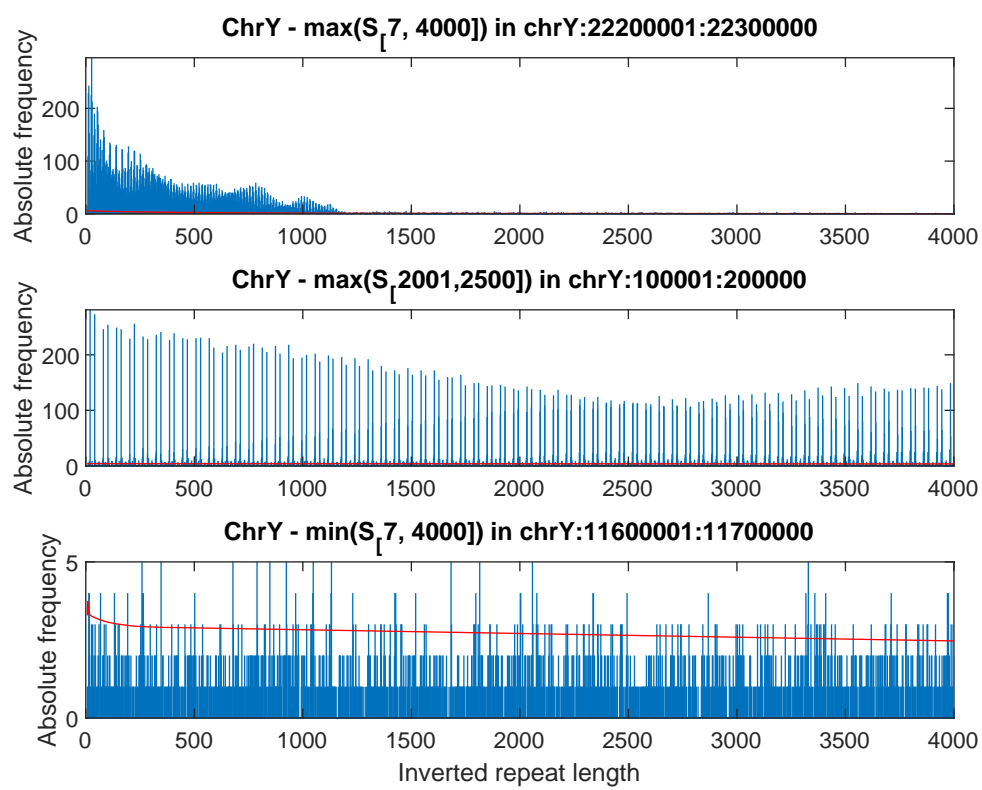

Supplement: Supplementary file 1 — Supplementary Material Details [file j_jib-2022-0052_suppl_001.zip › fig_dists_chrY.pdf]
